# Country Physicians

**Published:** 1886-11-20

**Authors:** 


### COUNTRY PHYSICIANS: THE COLCHESTER IMBROGLIO.

The Times are out of Joint.

"TEMPORA mutantur, et nos in illis mutamur." Railways have ruined country physicians. This is not offered as a translation, either free or literal, but is merely a more or less trite reflection occasioned by the changes of the times as they affect the medical world at Colchester and elsewhere. It is much to be feared, perhaps hardly to be regretted, that the days of Dr. Fillgrave and his yellow coach are quite numbered. Ever since that morning, big with fate, when Sir Roger Scatcherd, the erewhile stonemason, ordered the magnate of county medicine to be "put under the pump," the degenerate Thornes, who make up their own pills and label their own physic-bottles, have gradually been getting it all their own way. "O tempora, O mores—," we will not finish the quotation. What is to be done? We cannot resuscitate the past. The dodo is extinct, and so is the megatherium. True, if you give us but a coccygeal vertebra, or even a wing of the sphenoid, we can reproduce their skeletons in all their pristine completeness. But what is the use of a skeleton, even if it be the skeleton of a country physician? It cannot look wise, it cannot write a prescription, it cannot so much as shake its head, and say, "Ah!" or, "Oh!" or, "Whilst there is life there is hope, madam."

A White Elephant.

The story of Colchester and its physicians is that of many a similar town. Time was when what is called a pure physician could obtain work enough and fees enough to maintain him in that dignified respectability which is the delight of every Briton, especially if he belongs to "the Medical Faculty." This is no longer the case. The last of the Colchester physicians has resigned, and his office has for some time actually been going a-begging. It is a white elephant; nobody wants it. Not that there is any lack of physicians in the country. On the contrary, they are as plentiful as briefless barristers, and almost as impecunious. But a pure physician—whatever that may be—cannot live in Colchester, and that is the crux of the whole difficulty. The question now is this: Since Colchester cannot maintain a *pure* physician, ought it really to have a physician at all? That question the Infirmary authorities have been debating for some time, and have finally answered in the negative. They have followed up their decision by placing the medical as well as the surgical in-patients under the charge of the visiting surgeons. That is to say, they have declared by their action that there is no difference between medicine and surgery, or between physicians and surgeons; that medicine is one and indivisible, and that the modern idea of specialisation is a mistake and a superfluity.

The Surgeons in Error.

We cannot but express our emphatic dissent from such a position. The distinction between medicine and surgery, between the surgeon and the physician, is as old as science and as universal as art. What everybody believes, and believes always and believes everywhere, is not to be lightly set aside, even on the authority of the surgeons of Colchester. The Infirmary authorities have made a mistake, and have allowed themselves to be led in a wrong direction. They should retrace their steps with as little delay as possible. Their surgical staff have taken a false step, and if they have any *esprit de corps*, any regard for the honour of their profession, they will reconsider their position. Colchester has surgeons for its surgical patients, it should also have physicians for its medical patients. The surgeons are presumably in family practice. Why should the physicians not also be in family practice? Nowadays, what is called a pure physician is hardly known even in London. Some of the most eminent of the Edinburgh physicians—men whose names have made medicine famous in the remotest parts of the world—were family doctors to the end of their days. In America all the family doctors who are University graduates are called physicians, and properly so. Many hospital physicians in the country, if not in London, are in general practice, and glad so to be. Why should Colchester hold fast by an antiquated tradition, to the injury of its hospital, and the degradation of the status of its medical practitioners?

A Physician Wanted; and Found.

The proper course now to pursue is to appoint the local practitioner who is most trusted by his professional brethren to the post of physician, with all its honours and privileges, and let him retain the best portion of his family practice. If there be no practitioner who, by the general consent of his brethren and the public, is worthy of such an appointment, a suitable graduate should be advertised for, who should be allowed to enter upon general practice. There is many a man of brilliant University distinction who, under such circumstances, would gladly seek the appointment; who would apply himself with ardour to the scientific practice of his profession, both among hospital and private patients; who by virtue of his hospital status would demand and receive higher fees than those of the average practitioner, and who would raise the level of the whole profession, both in emolument and social position. There is no fear that such a man would poach on the preserves of his neighbours. He would make preserves for himself, and his influence would tend to the elevation of every other practitioner. We strongly counsel the profession and the infirmary managers to bring themselves into accord with the rest of the world, and with the scientific tendencies of the times.

